# Improvement of the qmosRT-PCR Assay and Its Application for the Detection and Quantitation of the Three Serotypes of the Novel Oral Polio Vaccine in Stool Samples

**DOI:** 10.3390/vaccines11111729

**Published:** 2023-11-19

**Authors:** Hasmik Manukyan, Erman Tritama, Rahnuma Wahid, Jennifer Anstadt, John Konz, Konstantin Chumakov, Majid Laassri

**Affiliations:** 1 Division of Viral Products, Center for Biologics Evaluation and Research, US Food and Drug Administration, 10903 New Hampshire Avenue, Silver Spring, MD 20993, USA; 2 Research and Development Division, PT. BioFarma, Bandung 40161, Indonesia; 3 Center for Vaccine Innovation and Access, PATH, Seattle, WA 98121, USAjanstadt@path.org (J.A.);

**Keywords:** clinical samples, OPV, nOPV, virus excretion, vaccines, poliovirus

## Abstract

Recently, genetically stable novel OPVs (nOPV) were developed by modifying the genomes of Sabin viruses of conventional OPVs to reduce the risk of reversion to neurovirulence and therefore the risk of generating circulating vaccine-derived polioviruses. There is a need for specific and sensitive methods for the identification and quantification of nOPV viruses individually and in mixtures for clinical trials and potentially for manufacturing quality control and environmental surveillance. In this communication, we evaluated and improved the quantitative multiplex one-step reverse transcriptase polymerase chain reaction (qmosRT-PCR) assay for the identification and quantification of nOPV viruses in samples with different formulations and virus concentrations and in virus-spiked stool samples. The assay was able to specifically identify at least 1 log_10_ CCID_50_/mL of each serotype in the presence of the two other serotypes at high concentrations (6–7 log_10_ CCID_50_/mL) in the same sample. In addition, the lowest viral concentration that the assay was able to detect in stool samples was 17 CCID_50_/mL for nOPV1 and nOPV2 viruses and 6 CCID_50_/mL for nOPV3. We also found high correlation between the expected and observed (by qmosRT-PCR) concentrations of spiked viruses in stool samples for all three nOPV viruses, with R-squared values above 0.95. The analysis of samples collected from an nOPV2 clinical trial showed that 100% of poliovirus type 2 was detected and few samples showed the presence of type 1 and 3 residuals from previous vaccinations with bOPV (at least 4 weeks prior vaccination with nOPV2), confirming the high sensitivity of the method. The qmosRT-PCR was specific and sensitive for the simultaneous identification and quantification of all three nOPV viruses. It can be used as an identity test during the nOPV manufacturing process and in evaluation of virus excretion in nOPV clinical trials.

## 1. Introduction

The live oral poliovirus vaccines (OPVs) are very effective against poliomyelitis and induce both humoral and mucosal immunity. They are produced from poliovirus Sabin strains that are inherently unstable and can revert to a neurovirulent phenotype during their replication in cell cultures or in vaccinees, resulting in rare cases of vaccine-associated paralytic poliomyelitis (VAPP) in vaccine recipients or in their contacts, in addition to the emergence of infectious circulating vaccine-derived polioviruses (cVDPV) that cause small outbreaks of paralytic poliomyelitis [1].

In September 2015 and October 2019, wild type 2 and 3 polioviruses were declared eradicated [2,3]. To alleviate the risk of VAPP and the emergence of cVDPV2, in April 2016, the routine vaccination with a trivalent OPV was exchanged with a bivalent OPV (bOPV) that contains only the Sabin 1 and 3 viruses. To prevent gaps in immunity to type 2 poliovirus, the schedule was supplemented with at least one dose of trivalent inactivated poliovirus vaccine (IPV) [4,5]. IPV, although effective at preventing poliovirus disease in recipients, is known to stimulate suboptimal mucosal immunity that does not prevent poliovirus transmission [6,7]. As a consequence, a vaccine recipient of bOPV with IPV can be infected with type 2 poliovirus and spread the virus [8,9,10]. Since the vaccination schedule change, cVDPV2 outbreaks have amplified dramatically [11]. To contain these outbreaks, monovalent OPV2 (mOPV2) was implemented, which resulted in seeding other new cVDPV2 outbreaks [12,13].

The most important attenuating mutations in Sabin genomes are situated in domain V of the 5′ untranslated region (UTR): C_472_U mutation and A_537_G in the Sabin 3 virus [14,15], G_481_A mutation in the Sabin 2 virus [16], and A_480_G and C_525_U mutations in the Sabin 1 virus [17]. They restore their virulence through virus replication in animals or in cell cultures. The level of attenuation is determined by the thermal stability of domain V: the high stability of the hairpin structure correlates well with neurovirulence. A_481_G reversion in the Sabin 2 virus emerges quickly, within a week after vaccination [18,19].

Recently, a novel OPV2 vaccine candidate (nOPV2-c1) was developed [20]. The candidate featured a V domain genetically modified by changing all U–G and some C–G pairs of nucleotides with strong U–A pairs [21]. As a result, the structure of domain V remains genetically stable, preserving the attenuation level of the virus but reducing the likelihood of mutations that increase neurovirulence. In addition, the nOPV2-c1 cis-acting replicative element (cre) was displaced from the center of the genome to the 5′ UTR, preventing the replacement of the genetically stabilized domain V through recombination. nOPV2-c1 also contains D_53_N and K_38_R amino acid substitutions in the 3D protein to increase replication fidelity and decrease the recombination degree, respectively.

nOPV strains of type 1 and type 3 were also developed using a strategy similar to nOPV2. nOPV1 and 3 were made using the genome of the nOPV2-c1 as a backbone; the capsid precursor P1 region of nOPV2-c1 was replaced with the P1 of Sabin-strains type 1 and 3 [22].

Poliovirus monitoring and surveillance in clinical and environmental samples is a vital part of the polio eradication effort. For clinical trials, evaluation of the excretion of vaccine polioviruses is used for confirming vaccine take, understanding the duration and extent of shedding, and assessment of the vaccine-induced mucosal immunity following challenge with OPV strains. Thus, methods to quantitatively evaluate the excretion of poliovirus are critical for conducting clinical trials. In this paper, we improve and evaluate the qmosRT-PCR method for the evaluation of nOPV shedding, including for multivalent mixtures. In addition, we present results that could support the use of the method for identity and impurity assessment during manufacturing release testing.

## 2. Materials and Methods

### 2.1. Vaccine Viruses and Clinical Samples

The US poliovirus vaccine references (GenBank accession numbers: AY184219 for Sabin 1 strain, AY184220 for Sabin 2 strain, and AY184221 for Sabin 3 strain) served as positive controls for each run of the qmosRT-PCR assay.

Three serotypes of nOPV (nOPV2-c1, nOPV1, and nOPV3) were kindly supplied by BioFarma (Indonesia) and were used for spiking in stool supernatant and for preparation of the qmosRT-PCR assay standard reference.

The coded, de-identified clinical stool samples used in this study were provided by the CDC and FIDEC from clinical trials of nOPV2 [23].

### 2.2. Primers and TaqMan Oligoprobes Used for OPV/nOPV Serotype Identification and Quantification

The unique primers for each of the three serotypes of oral poliovirus vaccine (OPV) were described previously [24]. They were selected from the RNA sequences of the capsid precursor protein region (P1) that are specific for each of the OPV strains and are missing in other enterovirus genomes. This set of primers was modified to detect and quantify nOPV and OPV strains even if they were present as mixtures in the same samples with large concentration differences. The type 1 and 3 reverse primer locations were changed to reduce their amplicon sizes to improve the sensitivity of the assay and to reduce the sensitivity difference between the detection of the three serotypes. The PCR amplification with this new set of primers resulted in DNA amplicons of 92, 122, and 69 base pairs long for serotypes 1, 2, and 3, respectively (Table 1). The primers were purchased from Integrated DNA Technologies, Inc. (Coralville, IA, USA), and TaqMan oligoprobes were purchased from Thermo Fisher Scientific (South San Francisco, CA, USA).

### 2.3. Extraction of Viral RNA

Total RNA was isolated from stool supernatants and from poliovirus-infected cell cultures using the QIAamp Viral RNA Mini Kit (QIAGEN, Chatsworth, CA, USA) in accordance with the manufacturer’s protocol. The obtained RNA was eluted in DEPC-treated water solution and stored at −80 °C.

### 2.4. qmosRT-PCR Amplification

The qmosRT-PCR reaction was prepared as described previously [24] with some modifications. In brief, qmosRT-PCR reactions were prepared in Micro 96-well Plates (Thermo Fisher Scientific, South San Francisco, CA, USA) in a final volume of 20 μL using 2 μL of viral RNA and the QuantiNova Multiplex RT-PCR Kit (QIAGEN, Valencia, CA, USA). Oligonucleotide TaqMan probes were used at a final concentration of 25 nM, each with the three pairs of primers (Table 1) at a concentration of 0.8 μM each. The qmosRT-PCR run was performed using a Quantitative PCR Machine ViiA7 (Thermo Fisher Scientific, South San Francisco, CA, USA) at the following PCR conditions: one cycle incubation for 10 min at 50 °C and 2 min at 95 °C, followed by 45 cycles, each consisting of 5 s at 95 °C, 10 s at 50 °C, and 30 s at 60 °C.

### 2.5. Sensitivity of the qmosRT-PCR Assay

The nOPV1, nOPV2, and nOPV3 viruses were propagated in HEp-2C cells and titrated using the WHO standard CCID_50_ assay [25,26], which generated results that were expressed in CCID_50_ (cell culture infectious dose 50%) per milliliter; samples with different concentrations and combinations of viruses were prepared and subjected to RNA extraction and qmosRT-PCR analysis in three replicates as described above.

### 2.6. Spike of nOPV2 and Trivalent nOPV in Stool Supernatant

Stool supernatant was prepared as described previously [19]. In brief, ten grams of stool were vortexed in 100 mL of DMEM (Dulbecco’s Modified Eagle Medium) and centrifuged at 350× *g* for 15 min. The supernatants were then aliquoted and stored at −80 °C. The stool supernatant was confirmed to be free from poliovirus using Illumina sequencing [27] and qmosRT-PCR [24] assays.

## 3. Results

### 3.1. Evaluation of the Specificity and Sensitivity of the Simultaneous Detection and Quantification of Each nOPV Strain in the Presence of High Concentrations of the Two Other Serotypes

Previously, we have developed qmosRT-PCR for the detection of OPV serotypes in clinical samples [24]. The method was able to detect and quantify nOPV serotypes with the same sensitivity and specificity as the primers and the probes also match the genomes of the nOPV strains. The analysis of spiked samples containing different concentrations and combinations of the viruses demonstrated that the method was more sensitive for nOPV2 virus detection than for detection of the nOPV1 and 3 viruses. This influenced the detection of nOPV1 and nOPV3 present in low concentrations (2–3 log_10_ CCID_50_/mL) when mixed with other nOPV types at high concentrations (6–7 log_10_ CCID_50_/mL) (Table 2).

To improve the qmosRT-PCR assay, we redesigned reverse primers for the type 1 and 3 strains (Table 1) by reducing the PCR amplicon sizes and thereby increasing the sensitivity of detection for the type 1 and 3 nOPV viruses.

To evaluate the specificity of the qmosRT-PCR method with the new primers for the simultaneous detection of nOPV viruses, we prepared samples with different combination of nOPV viruses and subjected them to qmosRT-PCR analysis using a standard reference: a mixture of RNA extracted from all three nOPV strains with known titers expressed in CCID_50_/mL. This was performed to allow calculation of the concentration of the test samples in CCID_50_/mL units. The results are presented in Table 3. These results showed no cross amplification between the nOPV viruses and no interference between the qPCR dyes, demonstrating that the method is specific for the identification and quantification of each nOPV serotype.

To demonstrate that the method was able to a detect low amount of one nOPV strain in the presence of high quantities of two other strains, we analyzed samples with different concentrations and combinations of the nOPV viruses. The results are presented in Table 4. These results show that all three serotypes were detectable when present at concentrations of 1 log_10_ CCID_50_/mL or higher even when in the presence of high concentrations (6–7 log_10_ CCID_50_/mL) of the other types. These data suggest that the accuracy for nOPV1 and nOPV3 may be better than the accuracy for nOPV2 at the low end of the operating range but that it is similar at concentrations of 3 log_10_ CCID_50_/mL and higher.

### 3.2. Analysis of Monovalent nOPV2-c1 and Trivalent nOPV Spiked in Stool Supernatant

The experiment was conducted to evaluate the ability of qmosRT-PCR to detect nOPV2-c1 in stool samples. A stool supernatant that was confirmed to be free from poliovirus was used to spike monovalent nOPV2-c1 virus with three-fold serial dilutions from 5.55 to 0.30 log_10_ CCID_50_/mL. The same spiking samples were prepared in duplicate (sets 1 and 2), with each set tested in triplicate. The results from the six replicates are summarized in Table 5. The method was able to detect at least 18 CCID_50_/mL of nOPV2-c1 in stool samples.

**Figure 1:**
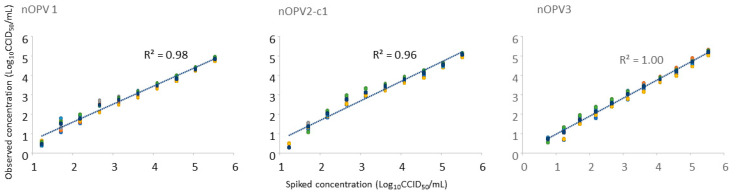
Evaluation of the correlation between the expected and observed concentrations of the trivalent nOPV spiked into stool supernatants analyzed using the qmosRT-PCR assay.

The nOPV1, 2, and 3 viruses were combined with a 7 log_10_ CCID_50_/mL concentration of each type and spiked with three-fold serial dilutions (from 5.52 to 0.27 log_10_ CCID_50_/mL) in stool supernatant that was free from poliovirus. The same spiked samples were prepared in duplicate (sets 1 and 2). Each set was tested in triplicate via qmosRT-PCR. The results of the six replicates are summarized in Table 5. The lowest viral concentration that the method was able to detect in stool samples was 17 CCID_50_/mL for nOPV1 and nOPV2 and 6 CCID_50_/mL for nOPV3. In addition, the expected concentrations of the spiked samples correlated well with the observed concentrations, with R-squared values of 0.98 for nOPV1, 0.96 for nOPV2-c1, and 1.00 for nOPV3 (Figure 1). We concluded that the method can be used for the evaluation of the stool excretion of nOPV viruses in clinical trials.

### 3.3. Analysis of Stool Samples from nOPV2 Clinical Trials

Twenty-six stool extracts were collected from infants following vaccination with bivalent OPV (containing only types 1 and 3) and then nOPV2-c1 (*n* = 26). These extracts were analyzed using qmosRT-PCR as described above. The results are shown in Table 6. All 26 nOPV2-containing samples were positive by the qmosRT-PCR assay for type 2 virus. Some samples were identified as containing residual Sabin-1 or -3 viruses from previous bOPV vaccinations from more than 4 weeks earlier than the vaccination with nOPV2-c1.

## 4. Discussion

To help the ongoing poliovirus eradication efforts, genetically stabilized novel OPV vaccine candidates (nOPV1, nOPV2, and nOPV3) have been developed [20,22]. In November 2020, the monovalent nOPV2-c1 was approved by WHO for emergency use [28]. This permits the vaccine to be used in countries experiencing cVDPV2 outbreaks. Since March 2021, over 600 million doses of nOPV2-c1 have been administrated. The available clinical and field surveillance data prove that nOPV2 is safe and effective and has a very low risk of changing to a form that causes poliomyelitis paralysis in low immunity settings in comparison with monovalent oral polio vaccine type 2 (mOPV2) [29].

With the pending clinical development of multivalent nOPV mixtures, a sensitive, specific, quantitative, and high-throughput assay is needed to detect poliovirus mixtures in clinical samples. Such methods may also be useful to monitor environmental samples or the manufacturing process of live and inactivated poliovirus vaccines.

The current methodology for the clinical evaluation of nOPV and comparator OPV strains employs a non-quantitative PCR for the intratypic differentiation of strains, typically followed by measurement of the infectious titer (CCID_50_) of samples that are positive for a single type [23]. Although effective for the development of monovalent nOPVs, this approach does not allow for quantitative evaluation of shedding when multiple types are present in most samples, as will be the case for development of multivalent nOPVs.

Recently we have developed a qmosRT-PCR method for the direct identification and quantitation of all three Sabin OPV strains in clinical and environmental specimens [24]. The method was shown to be rapid, sensitive, and specific for the identification and quantification of all three serotypes of Sabin viruses.

In this communication, we further improved the sensitivity of the primer/probe sets for types 1 and 3 and evaluated the assay as an alternative to the qPCR method for detecting Sabin-related strains [30]. In the qmosRT-PCR, concentrations of 1 CCID_50_/mL or higher were detected for each serotype even in presence of a high amount (6–7 log_10_ CCID_50_/mL) of the two other strains of the nOPV. The method also was able to identify each of the nOPV strains spiked into stool samples when present at approximately 1 log_10_ CCID_50_/mL, indicating that the method has excellent sensitivity for the identification of nOPV viruses in clinical samples.

We found strong correlations between the expected concentrations of the spiked nOPV in stool samples and the observed concentrations for all the three nOPV viruses; however, the values estimated from the standard curve were consistently lower than the calculated values from the spike-in (Table 5 and Figure 1). These results suggest that the PCR amplification is impacted by components of the stool extract. Assessing the consistency of this bias between stools may be beneficial to fully understand the impact on the accuracy of quantitation.

In addition, the assay detected nOPV2 in all 26 stool extracts from a recent nOPV2 study. The method was also able to identify some trace amounts of Sabin-1 or -3 residuals from a previous bOPV vaccination (from more than 4 weeks before the recipients received the nOPV2 dose); however, it is unknown if sample degradation may have impacted the sensitivity of the qmosRT-PCR.

## 5. Conclusions

The qmosRT-PCR method described in this communication offers a simple and rapid substitute to existing PCR or conventional cell-culture-based assays to identify and quantify either individual or all three serotypes of nOPV/OPV viruses. The qmosRT-PCR method could be useful for the detection and quantification of polioviruses in the stool specimens collected during clinical trials of new poliovirus vaccines (nOPV) and during routine clinical and environmental poliovirus surveillance. The qmosRT-PCR assay could also be used during the production of poliovirus vaccines to identify the presence and amounts of the three serotypes of vaccine polioviruses. The assay is also suitable for automation, which would further simplify large scale analyses, improve consistency, and save time, labor, and cost.

## Figures and Tables

**Table 1:** Primers and TaqMan probes for identification and quantification of the three nOPV/OPV serotypes.

nOPV/OPV Serotype	Oligo Name	Location in nOPV Genome	Sequence 5′-->3′	Size (nt)	Tm (°C, Basic)	Amplicon Size (bp)
1	2771Sab1F	2831-2849	CAGCTTCCACCAAGAATAA	19	43	92
	Sab1-2962R	2922-2904	GAAGAACTCCAATTTCCTC	19	47	
	Sab1-FAM	2863-2877	FAM-ACAGTGTGGAAGATC-NFQ	15		
2	2682TqS2F	2748-2763	CCAGAGACGAACGCGA	16	49	122
	2802TqS2R	2869-2852	AAACCGAAAACAATCTGC	18	44	
	Sab2-VIC	2772-2787	VIC-CACGGTTGAGTCATTC-NFQ	16		
3	1411TqS3F	1479-1497	GGGAAAATTTTACTCCCAA	19	45	69
	Sab3-1487R	1547-1527	ACTGGGCAGAACTCTCTTTTT	21	51	
	Sab3-NED	1510-1524	NED-AACGCAGTAACATCC-NFQ	15		

**Table 2:** Analysis of samples containing different concentrations and combinations of nOPV strains using the qmosRT-PCR assay.

nOPV Serotypes	Expected Titers *	qmosRT-PCR Result ^γ^		Expected Titers *	qmosRT-PCR Result ^γ^	
		Ct	log_10_ CCID_50_/mL		Ct	log_10_ CCID_50_/mL
1	3	34.96	1.35 ± 0.33	3	**UD**	
2	3	26.15	3.51 ± 0.08	7	15.95	6.52 ± 0.07
3	7	14.89	6.79 ± 0.08	7	17.06	6.22 ± 0.05
1	3	25.55	3.81 ± 0.03	4	32.34	2.04 ± 0.09
2	7	14.42	6.97 ± 0.06	7	15.83	6.56 ± 0.03
3	3	28.51	3.21 ± 0.18	7	17.04	6.23 ± 0.01
1	7	12.05	7.34 ± 0.04	2	**UD**	
2	3	25.78	3.62 ± 0.03	6	18.93	5.64 ± 0.05
3	3	28.62	3.18 ± 0.09	6	19.95	5.46 ± 0.05
1	7	12.04	7.35 ± 0.02	3	29.62	2.75 ± 0.01
2	7	14.00	7.1 ± 0.03	3	28.20	2.9 ± 0.01
3	3	**36.28**	1.16 ± 0.59	6	20.07	5.43 ± 0.05
1	7	12.08	7.33 ± 0.05	4	23.29	4.4 ± 0.11
2	3	26.89	3.29 ± 0.06	4	24.59	3.97 ± 0.07
3	7	15.12	6.73 ± 0.08	6	20.28	5.37 ± 0.09
1	3	**UD**		2	**UD**	
2	7	14.19	7.04 ± 0.02	2	33.06	1.46 ± 0.08
3	7	15.15	6.72 ± 0.13	6	20.19	5.4 ± 0.04
1	2	**UD**				
2	2	31.17	2.02 ± 0.07			
3	7	15.06	6.75 ± 0.04			
1	2	30.83	2.43 ± 0.02			
2	7	14.36	6.99 ± 0.02			
3	2	33.26	1.96 ± 0.07			
1	7	12.08	7.33 ± 0.02			
2	2	28.37	2.85 ± 0.09			
3	2	**40.54**	0.04 ± 0.47			

Note: *: Titers are expressed in log_10_ CCID_50_/mL. γ: Titer results of three repeats are presented as average ± STDEV of log values. UD: undetermined. Marked bold and underlined are Cts > 35 or those undetermined (indicating the virus was difficult to detect or not detected).

**Table 3:** Evaluation of the specificity of the qmosRT-PCR assay to detect and quantitate the three nOPV serotypes.

nOPV Serotypes	Expected Titer *	qmosRT-PCR Result ^γ^	
		Ct	log_10_ CCID_50_/mL
1	7	12.87	7.00 ± 0.02
2	7	16.58	6.90 ± 0.03
3	NT	UD	
1	7	12.75	7.03 ± 0.02
2	NT	UD	
3	7	14.79	6.91 ± 0.02
1	NT	UD	
2	7	17.28	6.70 ± 0.01
3	7	14.91	6.87 ± 0.02
1	7	12.84	7.01 ± 0.01
2	NT	UD	
3	NT	UD	
1	NT	UD	
2	7	17.89	6.52 ± 0.02
3	NT	UD	
1	NT	UD	
2	NT	UD	
3	7	15.04	6.84 ± 0.03
1	7	12.89	6.99 ± 0.03
2	7	16.55	6.91 ± 0.05
3	7	14.95	6.86 ± 0.03

Note: *: Titers are expressed in log_10_ CCID_50_/mL. γ: Titer results of three repeats are presented as average ± STDEV of log values. NT: No template, UD: Undetermined (>45 cycles).

**Table 4:** Analysis of samples contain different concentrations and combinations of nOPV strains using the improved qmosRT-PCR assay.

nOPV Serotypes	Expected Titer *	qmosRT-PCR Result	
		Ct	log_10_ CCID_50_/mL ^γ^
1	7	12.98	6.97 ± 0.01
2	7	16.63	6.89 ± 0.01
3	1	37.04	0.55 ± 0.38
1	7	12.55	7.09 ± 0.01
2	1	41.79	−0.25 ± 0.40
3	7	14.67	6.94 ± 0.05
1	1	33.43	0.83 ± 0.07
2	7	17.27	6.70 ± 0.00
3	7	14.98	6.85 ± 0.01
1	6	16.44	5.93 ± 0.02
2	6	20.48	5.78 ± 0.03
3	1	34.04	1.40 ± 0.33
1	6	16.24	5.99 ± 0.00
2	1	39.16	0.51 ± 0.39
3	6	18.14	5.94 ± 010
1	1	32.77	1.04 ± 0.12
2	6	21.07	5.61 ± 0.01
3	6	18.41	5.87 ± 0.13
1	6	16.36	5.95 ± 0.02
2	3	30.49	2.90 ± 0.03
3	1	35.09	1.01 ± 0.11
1	1	32.44	1.13 ± 0.03
2	6	22.47	5.21 ± 0.03
3	3	28.54	2.92 ± 012
1	3	25.82	3.11 ± 0.04
2	1	37.46	0.91 ± 0.17
3	6	18.56	5.82 ± 0.09

Note: *: Titers are expressed in log_10_ CCID_50_/mL. γ: Titer results of three repeats are presented as average ± STDEV of log values.

**Table 5:** Results of the qmosRT-PCR analysis of trivalent nOPV and monovalent nOPV2-C1 viruses spiked into stool supernatant.

Trivalent nOPV				Monovalent nOPV2-C1	
Expected Titers *	nOPV1 Titers ^γ^ (Average Ct)	nOPV2-C1 Titers ^γ^ (Average Ct)	nOPV3 Titers ^γ^ (Average Ct)	Expected Titers *	nOPV2-C1 Titers ^γ^ (Average Ct)
5.52	4.86 ± 0.06 (22)	5.08 ± 0.07(22)	5.21 ± 0.10 (22)	5.55	4.11 ± 0.09 (26)
5.05	4.32 ± 0.08 (24)	4.55 ± 0.09 (24)	4.70 ± 0.14 (24)	5.08	3.60 ± 0.10 (27)
4.57	3.83 ± 0.10 (26)	4.11 ± 0.14 (25)	4.21 ± 0.13 (26)	4.60	3.17 ± 0.05 (28)
4.09	3.48 ± 0.11 (27)	3.81 ± 0.12 (26)	3.81 ± 0.12 (27)	4.12	2.83 ± 0.04 (29)
3.61	3.06 ± 0.15 (29)	3.45 ± 0.12 (27)	3.43 ± 0.15 (28)	3.64	2.54 ± 0.11 (31)
3.14	2.75 ± 0.16 (30)	3.11 ± 0.15(29)	3.03 ± 0.18 (30)	3.17	2.30 ± 0.12 (32)
2.66	2.49 ± 0.19 (30)	2.77 ± 0.19 (30)	2.57 ± 0.15 (31)	2.69	1.89 ± 0.13 (34)
2.18	1.77 ± 0.18 (33)	2.03 ± 0.14 (32)	2.12 ± 0.20 (33)	2.21	1.54 ± 0.24 (34)
1.71	1.51 ± 0.29 (34)	1.36 ± 0.16(35)	1.72 ± 0.20 (34)	1.74	1.05 ± 0.31 (35)
1.23	0.45 ± 0.12 (37)	0.29 ± 0.38 (38)	1.08 ± 0.28 (36)	1.26	1.09 ± 0.71 (38)
0.75			0.72 ± 0.13 (37)	0.78	
0.27				0.30	

Note: *: Expected titers are expressed in log_10_ CCID_50_/mL. Ct: cycle threshold. γ: qmosRT-PCR results of six replicates are expressed in Log_10_CCID_50_/mL and presented as average ± STDEV of log values.

**Table 6:** qmosRT-PCR analysis of stool samples collected from infant recipients of nOPV2-c1.

Sample Codes	Detection Results				Titers (log_10_ CCID_50_/mL)		
	Type 1	Type 2	Type 3	All Types	Type 1	Type 2	Type 3
2	Neg	Pos	Neg	2		2.74	
3	Neg	Pos	Neg	2		3.43	
4	Neg	Pos	Pos	2, 3		3.64	4.55
5	Neg	Pos	Neg	2		2.76	
6	Neg	Pos	Neg	2		2.15	
7	Neg	Pos	Neg	2		2.15	
8	Neg	Pos	Neg	2		1.3	
9	Neg	Pos	Neg	2		2.28	
10	Neg	Pos	Neg	2		2.71	
11	Neg	Pos	Neg	2		1.99	
12	Pos	Pos	Pos	1, 2, 3	−0.27	5.26	0.04
13	Pos	Pos	Pos	1, 2, 3	−0.34	4.09	0.61
14	Neg	Pos	Neg	2		2.46	
15	Neg	Pos	Neg	2		2.54	
16	Neg	Pos	Neg	2		5.11	
17	Neg	Pos	Neg	2		2.83	
18	Neg	Pos	Neg	2		2.69	
19	Neg	Pos	Neg	2		0.55	
20	Pos	Pos	Pos	1, 2, 3	1.85	2.47	0.11
21	Neg	Pos	Neg	2		2.95	
22	Neg	Pos	Neg	2		2.25	
23	Neg	Pos	Neg	2		5.45	
24	Neg	Pos	Neg	2		3.35	
25	Neg	Pos	Neg	2		3.43	
26	Neg	Pos	Neg	2		4.43	
27	Neg	Pos	Neg	2		4.77	

Note: NEG: qmosRT-PCR result negative. Pos: qmosRT-PCR result positive.

## Data Availability

All relevant data are within the paper.
